# Hospital outcomes of older people with cognitive impairment: An integrative review

**DOI:** 10.1002/gps.4919

**Published:** 2018-06-26

**Authors:** Carole Fogg, Peter Griffiths, Paul Meredith, Jackie Bridges

**Affiliations:** ^1^ Research and Innovation Portsmouth Hospitals NHS Trust Portsmouth UK; ^2^ National Institute of Health Research Collaboration for Leadership in Applied Health Research and Care Wessex UK; ^3^ School of Health Sciences and Social Work, Faculty of Science University of Portsmouth Portsmouth UK; ^4^ Faculty of Health Sciences University of Southampton Southampton UK

**Keywords:** cognitive dysfunction, dementia, integrative review, older people, outcomes, patient admission

## Abstract

**Objectives:**

To summarise existing knowledge of outcomes of older hospital patients with cognitive impairment, including the type and frequency of outcomes reported, and the additional risk experienced by this patient group.

**Methods:**

Integrative literature review. Health care literature databases, reports, and policy documents on key websites were systematically searched. Papers describing the outcomes of older people with cognitive impairment during hospitalisation and at discharge were analysed and summarised using integrative methods.

**Results:**

One hundred four articles were included. A range of outcomes were identified, including those occurring during hospitalisation and at discharge. Older people with a dementia diagnosis were at higher risk from death in hospital, nursing home admission, long lengths of stay, as well as intermediate outcomes such as delirium, falls, dehydration, reduction in nutritional status, decline in physical and cognitive function, and new infections in hospital. Fewer studies examined the relationship of all‐cause cognitive impairment with outcomes. Patient and carer experiences of hospital admission were often poor. Few studies collected data relating to hospital environment, eg, ward type or staffing levels, and acuity of illness was rarely described.

**Conclusions:**

Older people with cognitive impairment have a higher risk of a variety of negative outcomes in hospital. Prevalent intermediate outcomes suggest that changes in care processes are required to ensure maintenance of fundamental care provision and greater attention to patient safety in this vulnerable group. More research is required to understand the most appropriate ways of doing this and how changes in these care processes are best implemented to improve hospital outcomes.

**Key points**

- People with cognitive impairment have higher hospital mortality, a higher incidence of delirium, and longer hospital stays than patients with no cognitive impairment. In addition, intermediate outcomes such as dehydration, reduction in nutritional status, pain, decline in physical and cognitive function, and new infections in hospital may contribute to poorer final hospital outcomes, but have been less well described than in patients with a formal dementia diagnosis.
- It is important to identify whether older people in hospital have cognitive impairment or an existing diagnosis of dementia, to be aware of their increased susceptibility to adverse events in the hospital environment, and to provide appropriate surveillance for intermediate outcomes to prompt preventative action.
- Further studies of outcomes for people with cognitive impairment in hospital should consider the care environment, such as ward type, staffing, and episodes of being located outside their designated ward (“outlying”), as these factors may also influence outcomes.
- Development and use of core outcome sets for people with cognitive impairment is essential to fully understand and describe the patient journey both to evaluate day‐to‐day care, and for use in observational or interventional research.

## 1 INTRODUCTION

Between 25% and 40% of older people admitted to acute hospitals have been diagnosed with dementia, (eg, Alzheimer's disease, dementia syndrome according to Diagnostic and Statistical Manual of Mental Disorders (DSM) IV, etc.) or have evident cognitive impairment because of undiagnosed dementia or another cause.^1^, ^2^ People with dementia occupy approximately 25% of hospital beds in the UK, stay up to 6 times longer than other older patients, and have a greater risk of dying in hospital; however, outcomes for people with any cause of cognitive impairment (CI) are less well described.^3^, ^4^ Poor hospital outcomes, eg, death or new discharge to a residential home, may occur following a series of less frequently reported outcomes which patients with CI may be more likely to experience in hospital. These intermediate outcomes may be an appropriate focus of attention to target nursing and other care and treatment, as, to reduce these outcomes, we must first understand how and why these patients deteriorate in hospital and identify the specific risk factors at patient and hospital level. Knowing how day‐to‐day clinical and well‐being outcomes for patients with CI differ from those with no CI during hospitalisation could help us identify specific areas of prevention or care which could improve the journey, and therefore the final outcome, for these patients.

Dementia is significantly underdiagnosed in the community, and delirium and CI often pass undetected in hospital.^5^, ^6^ A full diagnostic assessment for dementia during an acute hospital admission for all older people is neither appropriate nor feasible. However, simple cognition screening tests can be used to detect CI, eg, the Abbreviated Mental Test Score for cognitive function or the Confusion Assessment Method for delirium.^7^, ^8^, ^9^ Studies of acutely hospitalised older people using systematically applied screening tests for CI have highlighted that a significant proportion do not have a dementia diagnosis, but patients with CI experience rates of hospital outcomes, eg, mortality, more similar to those of patients with dementia than patients with no CI.^10^, ^11^ Greater understanding of the outcomes of older people with various causes of CI should inform how we can improve care for the whole population at risk. There are currently no published reviews in this area.

This review aims to summarise existing available evidence about the outcomes of older patients with cognitive impairment admitted to hospital, specifically to establish which outcomes have been investigated, the additional risk of outcomes in people with CI, and factors that may influence outcomes.

## 2 METHODS

### 2.1 Integrative review method

Integrative review methodology enables inclusion of a broad range of study designs and nonresearch literature, eg, audits and theoretical perspectives.^12^, ^13^ The method summarises findings with mixed narrative and tabular presentation, identifies common themes in study results, and highlights inconsistencies, without numerical synthesis.

### 2.2 Data sources and search strategy

MEDLINE, Cumulative Index to Nursing and Allied Health Literature, PsycINFO and EMBASE, AgeInfo, and the Cochrane Library were searched. Terms used (eg, medical subject headings) to describe the population included (1) demographic group: “Aged, hospitalised”, “aged hospital patient”, aged, geriatric, senior; (2) clinical group: “cognition disorders”, dementia, “Alzheimer's disease”, “cognitive impairment”, “cogniti* impair*”, “cognitive defect” “delirium/dementia/amnestic, cognitive disorders”, “frontotemporal dementia”, “dementia vascular”, “dementia, multi‐infarction”, “Lewy body dementia”, “dementia, senile”; and (3) health service use group: “hospital admission”, hospital*. (See supplementary material). Additional evidence was retrieved by reviewing reference lists, forward citation searches, and searching websites of organisations focussing on the care of older people, eg, Age UK, British Geriatrics Society, Royal College of Nursing, Alzheimer's Society, and Alzheimer's UK.

### 2.3 Criteria for inclusion of evidence

Studies included were those which investigated (i) outcomes of older people with CI with a hospital admission as a main purpose of the study, or (ii) the contribution of CI to an outcome of interest related to hospitalisation, including other disease outcomes, surgical, or medical treatments, or (iii) outcomes of people with CI in intensive care units during hospital admission, where the outcomes occurred during hospitalisation or at discharge. The search was limited to articles published in the last 20 years (since 1997) as these will reflect contemporary service provision, care practices, and up‐to‐date methods of detecting dementia/CI. Studies which reported on outcomes of Emergency Department visits only, elective surgical patients, patients with delirium with no evidence of prior CI, and those taking place within specialised psychogeriatric units were excluded.

### 2.4 Evaluation of evidence

Titles and abstracts were screened for review aims. Full texts were obtained for potentially relevant articles and screened against eligibility criteria. Screening and data extraction was undertaken by a single reviewer, and decisions checked with a second reviewer in case of uncertainty. The relevance of all included studies was verified by 3 reviewers. As one of the purposes of this review was to understand which outcomes are being measured for this population in hospital, no formal quality assessment was performed to maintain inclusivity. Methodological issues, eg, the potential for bias, are indicated in text or tables where appropriate.

## 3 RESULTS

**Figure 1:**
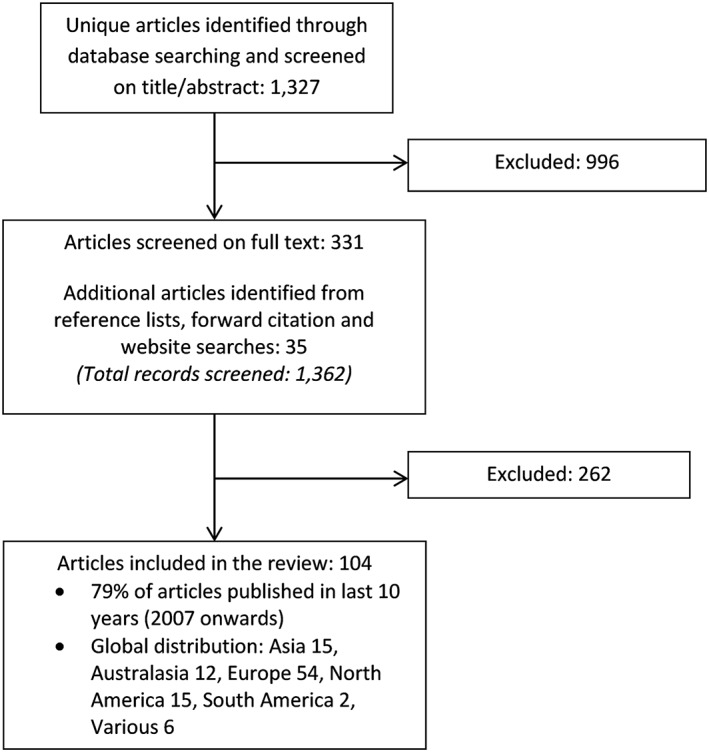
Selection of articles

One thousand sixty‐two records were identified from database searches, reference lists, and website searches. Following review of abstracts and full papers against eligibility criteria, 104 articles were included in the review (Figure 1). The median number of participants was 498 (range 4‐3 000 000), mostly of people aged ≥50. Participant cohorts included general inpatients, specific conditions, eg, heart failure or fractures, or with specific clinical interventions, eg, catheterisation. CI was defined in several ways, eg, of dementia diagnosis, cognitive spectrum disorder (delirium, dementia, or Abbreviated Mental Test <8), or other assessments, eg, Short Blessed Test.

The articles encompass a range of methodologies, eg, observational studies comparing patients with/without CI, studies in which cognitive status or dementia were evaluated as risk factors for specific outcomes, and qualitative studies and audits. A variety of outcomes were explored, not only in patients with dementia compared to those without but also in patients with measurable CI regardless of diagnosis. Associations between CI and outcomes were assessed using a variety of covariates, reflecting the study context and data sources available. Articles with more than 1 outcome are presented in the appropriate tables.

### 3.1 Clinical and patient‐centred outcomes during hospitalisation

#### 3.1.1 Patients' experiences of hospital admission

An integrative review summarising 24 papers on patient and carer experience concluded that people with dementia are stigmatised in hospitals, and acute care needs and tasks are prioritised over personalised care^14^ (Table 1). The UK National Audit of Dementia Care found that 17% of comments about patient care (collected via a carer questionnaire) described care negatively, and 9% expressed that patient did not receive care appropriate to their needs.^15^ Surveys estimate that around 60% of people with dementia are not treated with dignity or understanding whilst hospitalised, and the majority are frightened by the hospital environment.^3^ Reporting of negative experiences has been observed to follow a model, the “cycle of discontent”, in which poor communication and relationship building between staff and patients/carers lead to expectations not being met, subsequent cycles of identification of poor care and challenge to staff, further deterioration in the relationship, and ultimately reporting of poor experiences.^16^ It has been observed that there are many missed opportunities in hospitals to provide person‐centred care and enable a person with dementia to sustain personhood.^17^ No studies were found that discussed experiences of older patients with any cause of CI.

**Table 1:** Clinical and patient‐centred outcomes during hospitalisation^a^

Authors, year	Country	Population	Study design	Main results
Patients' experience of hospital admission				
Digby et al. 2016^14^	Various	Patients with dementia and their carers in the acute setting	Integrative review	People with dementia stigmatised in hospitals; acute care needs and tasks prioritised over personalised care; relatives/carers are not as involved in the patient's care or decisions regarding their relative as they could be.
Royal College of psychiatrists 2017^15^	UK	Patients with dementia in the acute setting	National audit	17% of comments about patient care collected via a carer questionnaire described care as generally poor, or alternative negative comment. 9% of comments expressed that the patient did not receive care appropriate to their needs.
Alzheimer's UK 2016^3^	UK	Carers of patients with dementia in the acute setting	Survey and freedom of information requests	Almost 60% of respondents felt the person with dementia they know was not treated with dignity or understanding while in hospital, 92% said hospital environments are frightening for the person with dementia.
Jurgens et al. 2012^16^	England	35 family carers of confused older patients	Qualitative interviews	Development of “cycle of discontent” model: Poor communication and relationship building between staff and patients/carers led to expectations from the patient/carer not being met, and subsequent cycles of identification of poor care by carers, challenge to staff, further deterioration in the relationship and reporting of poor experience occurring
Clisset et al. 2013^17^	UK	34 patients with dementia admitted to acute general medical, health care for older people, and orthopaedic wards, family carers and copatients	Non‐participant observations, qualitative interviews	Person‐centred care was observed, but there were more opportunities to sustain personhood, according to Kitwood's 5 domains of person‐centred care—Identity, inclusion, attachment, comfort, and occupation.
Behavioural and psychological symptoms of dementia (BPSD)				
Sampson et al. 2014^18^	UK	230 patients aged 70+ with dementia admitted to hospital for acute medical illness	Prospective cohort	The prevalence of BPSD symptoms in people with dementia in hospital rose from 62% at baseline, to 75% during the admission, with 43% being moderately/severely troubling to staff. The overall Behavioural pathology in Alzheimer disease scale (BEHAVE‐AD) score was in turn associated with an increase in mortality: aOR 1.11 [1.01–1.20], *P* = .022
Soto et al. 2012^19^	France	6299 patients with dementia admitted to an Alzheimer special acute care inpatient unit	Observational study	BPSD was the most frequent cause of complications, with agitation/aggressiveness representing 60% of BPSD events
Porock et al. 2015^20^	UK	34 patients admitted to acute hospital care, and 32 carers	Qualitative study — Interviews	Disruption in routine, for example, admission to hospital, has a negative impact on a person with dementia, and can trigger changes in behaviour as the patient attempts to gain control over their unfamiliar environment.
Malnutrition or dehydration				
Kagansky et al. 2005^21^	Israel	414 patients aged 75+ admitted to geriatric ward, including 107 patients with dementia	Prospective cohort	People with dementia were more likely to have a low MNA at admission: OR 3.85 [1.55–9.59], as well as laboratory indices of malnutrition such as albumin, transferrin, and the urea/creatinine ratio. The MNA score and the sub‐score related to dietary habits (MNA‐3) were significant predictors of death in hospital, with scores <7.5 increasing the risk of death 2.05‐fold.
Miller et al. 2006^22^	Australia	68 patients aged 70+ admitted to orthopaedic ward for lower limb fracture, 50% with cognitive impairment (as per short portable mental status questionnaire (SPMSQ))	Prospective cohort	Cognitively impaired participants and those without cognitive impairment consumed, mean (95% CI) respectively, 3661 kJ/day (3201, 4121) vs 4208 kJ/day (3798, 4619) and 38 g (33, 44) vs 47 g (41, 52) protein/day. Cognitively impaired participants consumed mean (95% CI) 48% (43, 53) of estimated total energy expenditure and 78% (69, 87) of estimated protein requirements
Royal College of psychiatrists, 2017^15^	UK	Patients with dementia in the acute setting.	National audit	24% of staff did not think that people with dementia had their nutritional needs met always or most of the time, and less than 75% of staff said that they could obtain finger foods or snacks between meals for patients with dementia.
Johnson et al. 2015^23^	Sweden	256 patients admitted to acute hospital care	Prospective cohort	Concentrated urine present in 16% of the patients, and more common in patients with confusion and/or dementia. 30‐day mortality was higher in patients with fluid retention compared to those who were euhydrated: 21% vs 8%, *P* < .03.
Functional or cognitive decline				
Hartley et al. 2017^24^	Various	Adults 65+ with acute admission to hospital and have information on dementia/cognitive scores on admission, with 54 637 patients available for quantitative synthesis	Systematic review and metaanalysis	Functional decline in hospitalised adults aged 65 and above is associated with cognitive impairment (RR 1.64 [1.45–1.86]), and a diagnosis of dementia (RR 1.36 [1.05–1.76])
Pedone et al. 2005^25^	Italy	9061 older patients admitted to hospital	Prospective cohort	During admission, 4% of patients with CI at admission and 1.3% of those without CI experienced functional decline: OR 2.4 [1.7–3.5], *P* < .001. Cognitive decline was strongly associated with an increased risk of functional decline: OR 16.0 [10.8–23.6], *P* < .001.
Incident delirium during hospitalisation				
Ryan et al. 2013^6^	Ireland	311 general hospital inpatients	Point prevalence study	Prevalence of delirium was higher in patients with pre‐existing dementia: 50.9% of delirious patients, OR 15.33, *P* < .001
Ahmed et al. 2014^26^	Various	2338 older medical inpatients systematic review and metaanalysis	Dementia increased risk of delirium: OR 6.62 [4.3–10.19]	
Sá Esteves et al. 2016^28^	Portugal	270 male patients aged 65+ admitted to a medical ward	Prospective cohort study	The rate of delirium was increased with people with dementia compared to those without: 29.5% vs 7.1%, *P* < .001
Travers et al. 2014^29^	Australia	493 patients aged 70+, with (*n* = 102) and without (*n* = 391) dementia	Prospective cohort study	Dementia increased the risk of developing delirium during hospitalisation, from 4.8% to 14.7%: OR 4.8, *P* < .001
Pendlebury et al. 2015^27^	UK	503 patients with acute admission to hospital (308 patients 65+ with covariate information)	Prospective cohort study	The risk of delirium on admission or during hospitalisation was increased by dementia OR 2.08 [1.10–3.93], *P* = .024 and low cognitive score (mini‐mental state examination (MMSE) and AMTS) OR 5.00 [2.50 to 9.99], *P* < .0001.
Franco et al. 2010^30^	Colombia	291 geriatric patients in medical wards	Nested casecontrol in prospective cohort	Median MMSE score 24.23 in patients who did not develop delirium during admission, vs 20.65 in those who did (*P* = .0001)
Bo et al. 2009^31^	Italy	252 patients 70+ with emergency admissions to hospital	Prospective cohort	Greater cognitive impairment associated with incident delirium (*P* < .001)
Wilson et al. 2005^32^	UK	100 patients aged 75+ admitted to an acute medical ward	Prospective cohort	Lower informant questionnaire on cognitive decline in the elderly was related to an increased incidence of delirium: OR 3.26 [1.18–9.04] *P* = .023
Voyer et al. 2006^33^	Canada	104 patients aged 65+ admitted to acute care	Prospective cohort	Prevalence of delirium increased with decreasing cognitive ability: Mild CI: 50%, moderate CI: 82%, severe CI: 86%
Muangpaisan et al. 2015^34^	Thailand	80 patients with fall‐related hip fracture	Prospective cohort	Modified informant questionnaire on cognitive decline in the elderly score significantly different between delirium and nondelirium groups: Median 3.5 vs 3.2, OR 4.5 [1.2–16.9] *P* = .024
Marcantonio et al. 2000^35^	USA	126 patients aged 65+ admitted emergently for hip fracture repair	Prospective cohort	Prefracture cognitive impairment was related to occurrence of delirium following surgery: RR 2.5 [1.6–3.9]
Wu et al. 2015^36^	China	130 patients aged 65+ attending hospital for hip fracture repair	Prospective cohort	Preoperative MMSE scores were negatively associated with higher incidences and greater severity of postoperative delirium: Median MMSE of 18.1 (delirium) vs 24.3, *P* < .001
Tanaka et al. 2016^37^	Japan	152 patients aged 70+ for proximal femoral fracture surgery	Prospective cohort	Dementia predictive of perioperative delirium: OR 3.55 [1.35–9.30]
Jackson et al. 2016^38^	Various	27 studies examining predictors of delirium	Systematic review	Hospital outcomes including mortality, institutionalisation, and length of stay for patients with delirium are also worse if there is pre‐existing psychiatric morbidity such as dementia.
Fong et al. 2012^39^	USA	771 persons with Alzheimer's disease in the community, of whom 367 were hospitalized	Prospective cohort	Incidence of delirium in hospital was 25% (*n* = 194). Patients with delirium had a higher risk of death within 1 year (15.5% (30/194) vs 9.2% (16/173))
Torpilliesi et al. 2010^40^	Italy	2340 patients admitted to a rehabilitation and aged care unit	Prospective cohort	Delirium superimposed on dementia (DSD) and poor functional status are stronger predictors than dementia alone of adverse clinical outcomes (length of stay, institutionalisation).
Avelino‐Silva et al. 2017^41^	Brazil	1409 acute hospital admissions of patients aged 60+	Prospective cohort	Of the 549 patients with dementia, 66.8% (*n* = 367) had DSD. DSD was independently associated with in hospital mortality, HR 2.14 [1.33–3.45] *P* = .002, whereas dementia alone was not.
Hsieh et al. 2015^42^	USA	260 patients aged 65+ with an acute admission to hospital	Prospective cohort	Dementia was associated with an increased risk of occurrence of least 1 episode of delirium during the first 3 days of admission in adults aged 65 and above, and subsequently increased the odds of unanticipated ICU admission or in‐hospital death: aOR 8.07 [1.91–34.14].
Adverse events and complications occurring in hospital				
Mecocci et al. 2005^43^	Italy	13 729 patients aged 65+ admitted to medical or geriatric wards	Prospective cohort	Cognitive impairment was found to be the most significant risk factor for (i) pressure ulcers: OR 4.9 [2.4–9.9], (ii) development of new faecal incontinence: OR 6.3 [3.0–13.0], (iii) urinary incontinence: OR 5.3 [2.3–12.0], (iv) falls: OR 1.6 [1.2–2.3].
Härlein et al. 2011^44^	Germany	9 246 patients aged 65+ admitted to 37 hospitals, with 1276 (13.8%) rated as cognitively impaired	Secondary analysis of point prevalence studies	Cognitive impairment leads to an increased risk of falls in hospital: 12.9% with CI vs 4.2% without CI; aOR 2.1 [1.7–2.7]
Chen et al. 2011^45^	Australia	408 patients aged 70+ admitted to hospital	Retrospective case control.	Dementia was significantly associated with recurrent falls. Recurrent fallers had significantly lower MMSE scores than single fallers and nonfallers (17.3 ± 6.7, 20.2 ± 6.2, 24.0 ± 5.1 respectively, *P* < .01) and a larger proportion of recurrent fallers had MMSE <18 than in the other 2 groups (54.1%, 34.4% and 10.8% respectively, *P* < .01). Patients with recurrent falls were more likely to have significantly lower scores in the “registration”, “attention and calculation”, “recall”, and “praxis” domains of the MMSE than single fallers.
Ferrari et al. 2012^46^	USA	233 patients aged 65+ with a documented inpatient fall	Retrospective descriptive study	Falls related to impulsive behaviour are more common in patients with cognitive impairment.
Tängman et al. 2010^47^	Sweden	223 patients admitted to a ward in a psychogeriatric hospital ward	Prospective fall registration study and case‐note review	91 (41%) of patients fell, with a total of 298 falls. More than 3 quarters of falls had 1 of the following precipitating factors: Being in hospital at night (between 9 pm and 7 am), having an acute disease or symptoms of disease and/or acute drug side effects
Tamiya et al. 2015^48^	Japan	817 with in‐hospital fracture, 3158 controls	Matched case: Control study (national inpatient database)	Increased risk of fractures in patients taking short‐acting benzodiazepine hypnotics, OR 1.43 [1.19–1.73]; *P* < .001, ultrashort‐acting non‐benzodiazepine hypnotics OR 1.66 [1.37–2.01]; *P* < .001, hydroxyzine, OR 1.45 [1.15–1.82]; *P* = .001, risperidone and perospirone, OR 1.37 [1.08–1.73]; *P* = .010.
Bail et al. 2013^49^	Australia	426 276 overnight hospital episodes in patients aged 50+, matched 1 patient with dementia: 4 patients without dementia	Retrospective cohort study	Hospitalised medical and surgical patients with dementia were at higher risk of 4 common complications than medical/surgical patients without dementia: (i) UTIs med: RR 1.79 [1.70–1.90], surg: RR 2.88 [2.45–3.40], (ii) pressure ulcers med: RR 1.61 [1.46–1.77] surg: RR 1.84 [1.46–1.31], (iii) pneumonia med: RR 1.37 [1.26–1.48] surg: RR 1.66 [1.36–2.02], (iv) delirium med: RR 2.83 [2.54–3.15] surg: RR 3.10 [2.31–4.15]. Medical patients were also at higher risk from sepsis RR 1.34 [1.15–1.57] and failure to rescue RR 1.24 [1.02–1.33].
Pendlebury et al. 2015^27^	UK	503 patients with acute admission to hospital (308 patients 65+ with covariate information)	Prospective cohort study	Prior dementia and low cognitive score is associated with incident delirium in hospital, and delirium in turn increased the risk of falls, (OR 4.55 [1.47–14.05], *P* = .008), incontinence of urine (OR 3.76 [2.15–6.58], *P* < .0001) incontinence of faeces (OR 3.49 [1.81–6.73], *P* = .0002) and catheterization (OR 5.08 [2.44–10.54], *P* < .0001).
Furlanetto et al. 2016^50^	Australia	100 patients aged 65+ with dementia/CI, ambulant and continent preadmission	Retrospective case‐note review	57% had either urinary or faecal incontinence (or both) at some point during admission, with 36% and 2% respectively had new incontinence at discharge
Kanagaratnam et al. 2017^51^	France	293 patients with dementia syndrome admitted to an acute geriatric care unit within a hospital	Prospective cohort	Polypharmacy (≥5 drugs/day) (OR: 4.0, 95% CI: 1.1–14.1) and dependence on at least 1 activity of daily living (ADL) (OR: 2.6, 95% CI: 1.1–6.5) were related with ADRs
Borenstein et al. 2013^52^	USA	214 adult Medicare beneficiaries admitted to hospital, mean age 75 years	Prospective cohort	Cognitive impairment is associated with an increase in hospital acquired infection, ADRs and length of stay >7 days) OR 2.32 [1.24–4.37]
Onder et al. 2002^53^	Italy	16 296 patients admitted to 81 hospitals (GIFA study)	Prospective surveys	An ADR was recorded in 232/4883 (4.8%) patients with cognitive impairment (AMT score < 7) and in 744/12 043 (6.2%) patients cognitively intact: aOR 0.70 [0.60–0.83]. However, neuropsychiatric complications were significantly increased in patients with CI (aOR 2.23 [1.40–3.54]).
Onder et al. 2003^54^	Italy	5734 patients aged 65+ admitted to 81 hospitals (GIFA study)	Prospective surveys	Patients with cognitive impairment had a lower risk of using inappropriate medication, as defined by the beers criteria: OR 0.77 [0.64–0.94]
Marengoni et al. 2011^55^	Italy	1332 patients aged 65+ admitted to general medicine or geriatric wards	Prospective cohort	Dementia on its own was associated with an increase in hospital mortality (OR 2.1 [1.0–4.5]). The addition of at least 1 adverse clinical event (defined as any acute clinical problem that newly occurred during hospitalisation, eg, delirium, urinary tract infection, fever, anaemia, pneumonia, electrolyte disorders, atrial fibrillation, heart failure or acute renal failure) had an additive effect on mortality, increasing the OR to 20.7 [6.9–61.9].
Watkin et al. 2012^56^	UK	710 patients aged 70+ with emergency medical admission	Prospective cohort	AEs were associated with mild/moderate CI (OR 3.61 [1.72–7.61], *P* = .01) and dementia (OR 2.18 [1.10–4.32], *P* = .03). AEs were not subsequently associated with mortality: Hazard ratio (HR) 1.01 [0.53–1.93], *P* = .596.
Shen et al. 2012^57^	Taiwan	41 672 patients 65+ with inpatient claim in health insurance database, including 3487 with dementia	Retrospective cohort	Patients with dementia have a higher risk of acute organ dysfunction (aOR 1.32 [1.19–1.46]) and severe sepsis (aOR 1.5 [1.32–1.69]).
Liao et al. 2015^58^	Taiwan	15 539 hospitalised patients with COPD, including 1406 with dementia	Retrospective matched cohort	Patients with chronic obstructive pulmonary disease (COPD) with dementia had increased mortality (aOR 1.38 [1.10–1.72]). This may partly be explained by the increased odds of severe sepsis (aOR 1.38 [1.10–1.72]) and acute respiratory dysfunction (aOR 1.39 [1.09–1.77]).
Frohnhofen et al. 2011^59^	Germany	1424 patients with COPD admitted to a geriatric ward, including 740 patients with dementia	Prospective cohort	Whereas 42% (287/684) of patients with no dementia were receiving no treatment for their COPD, 64% (195/307) of patients with moderate/severe dementia had no treatment (*P* < .01). Patients with dementia were also less likely to have lung function tests completed successfully: OR: 2.80 [1.18–6.60] for mild and OR 4.92 [2.03–11.91] for moderate to severe dementia.

a Papers reporting on 1 outcome are repeated as necessary in the other tables of this paper.

#### 3.1.2 Behavioural and psychological symptoms of dementia

The prevalence of behavioural and psychological symptoms of dementia (BPSD) symptoms in people with dementia in hospital rises during admission, likely because of unmet needs and distress, and a higher overall Behavioural Pathology in Alzheimer Disease Scale (BEHAVE‐AD score) (incorporating BPSD) associated with increased mortality.^18^ Behavioural and psychological symptoms of dementia have been identified as a frequent cause of complications in an Alzheimer Special Acute Care inpatient unit, with agitation and aggressiveness representing 60% of BPSD events.^19^ A qualitative study identified disruption in routine, eg, admission to hospital, triggering negative changes in behaviour as the person with dementia attempts to gain control over an unfamiliar environment.^20^

#### 3.1.3 Malnutrition or dehydration

Older people with dementia are more likely to have a low Mini‐Nutritional Assessment (MNA) score and laboratory indices indicating malnutrition at hospital admission, with overall MNA score and subscore related to dietary habits (MNA‐3) significant predictors of death in hospital.^21^ Of admitted patients who are already undernourished, those with CI are less likely to meet their required energy and protein intake, achieving <50% of total energy expenditure requirements.^22^ Organisational factors may contribute to decline in nutritional status through lack of availability of adequate nutrition. An audit revealed that only 76% of staff considered people with dementia had their nutritional needs met “always or most of the time”, and <75% of staff said that they could obtain snacks between meals for patients with dementia, who were unable to eat full meals at regular times.^15^ Fluid intake is also a key indicator of fundamental care in hospital. Assessment of renal conservation of water in older patients showed concentrated urine in 16% of patients, more commonly in patients with confusion and/or dementia, and was related to higher 30‐day mortality.^23^

#### 3.1.4 Functional or cognitive decline

A meta‐analysis identified functional decline (measured by activities of daily living (ADL), instrumental ADLs (IADL), Barthel index (BI), mobility, functional independence measure (FIM), or Rankin scale) in hospitalised adults aged ≥65 is independently associated with CI or a dementia diagnosis.^24^ Further cognitive decline during hospitalisation is associated with an increased risk of functional decline, defined as a loss of ability to perform 1 or more ADLs without help between admission and discharge.^25^

#### 3.1.5 Incident delirium during hospitalisation

The prevalence of delirium in general hospital patients is around 20%, and approximately half these patients have pre‐existing dementia.^6^ Although patients with dementia are more likely to have delirium at admission, dementia increases the likelihood of new‐onset delirium (or “delirium superimposed on dementia” (DSD)) during hospitalisation.^26^, ^27^, ^28^, ^29^ Regardless of a dementia diagnosis, lower cognitive scores are associated with increased occurrence of delirium in hospital, and symptoms of greater severity, eg, disordered attention, orientation, thought organisation, and memory.^27^, ^30^, ^31^, ^32^, ^33^ Cognitive impairment and dementia are predictive of delirium occurring prior to or following surgery for fractures of the hip or proximal femur.^34^, ^35^, ^36^, ^37^ Hospital outcomes including mortality, institutionalisation, and length of stay for patients with delirium are worse with pre‐existing dementia.^38^, ^39^, ^40^, ^41^ Dementia was associated with an increased risk of least 1 episode of delirium during the first 3 days of admission in adults aged ≥65, and increased the odds of unanticipated ICU admission or in‐hospital death.^42^

#### 3.1.6 Adverse events and complications occurring in hospital

Events occurring during hospitalisation, eg, urinary tract infections (UTI), pneumonia, or gastroenteritis (hospital‐acquired infections (HAI)), pressure ulcers (PU), adverse drug reactions (ADR) falls, and fractures impair recovery by reducing mobility, functional ability, and nutritional status, increase care required, and extend hospitalisation. Cognitive impairment or dementia leads to an increased risk of falls in hospital,^43^, ^44^ including recurrent falls^45^ and falls related to impulsive behaviour.^46^ In addition, factors identified in >75% of falls in patients with dementia included being in hospital at night, acute disease or symptoms of disease, and/or acute drug side effects.^47^ Falls may result in fractures, which delay recovery and lengthen hospitalisation. Occurrence of fractures in patients with dementia is associated with hypnotic medicines, specifically short‐acting benzodiazepine hypnotics, ultrashort‐acting nonbenzodiazepine hypnotics, hydroxyzine, risperidone, and perospirone.^48^ Both medical and surgical inpatients with dementia are at higher risk of 4 common complications, UTIs, PUs, pneumonia, and delirium, and medical patients are also at increased risk from sepsis and “failure to rescue”.^49^ Pressure ulcers are also more common in patients with CI.^27^ Cognitive impairment was shown to be the most significant risk factor for developing urinary and faecal incontinence,^43^ with 36% and 2% new incontinence at discharge respectively.^50^

Polypharmacy (≥5 drugs/day) and dependence for at least 1 ADL were related to occurrence of at least 1 ADR in inpatients with dementia.^51^ Cognitive impairment in older people is associated with increased HAIs, ADRs, and length of stay ≥7 days^52^ Adverse drug reactions may be less frequently reported in patients with CI, because of reduced ability to recognise and communicate side effects, leading to unsafe care.^53^ However, older patients with CI may be less likely to use inappropriate medication (as per Beers criteria), thus reducing ADR reporting in this group.^54^ A study exploring the relationship of adverse clinical events (ie, any acute clinical problem that occurs newly during hospitalisation) and mortality in patients with dementia showed at least 1 adverse clinical event (eg, electrolyte disorders, hypertensive crisis, fractures, or infections) increased the risk of death 10‐fold.^55^ Mild/moderate CI was associated with adverse events defined as “incidents” (eg, following an unintended “accident” in hospital such as a slip or trip, medication error, or staff miscommunication), but not subsequent mortality.^56^

Inpatients with dementia have a higher risk of acute organ dysfunction and severe sepsis, particularly patients with comorbidities such as chronic obstructive pulmonary disease (COPD).^57^, ^58^ Inpatients with COPD and dementia were less likely to be receiving treatment for COPD and to have their lung function assessed, suggesting that undertreatment could contribute to poorer outcomes.^59^

### 3.2 Differences in care during hospitalisation

#### 3.2.1 “Outlying” and bed moves

Pressures on hospital beds lead to older people not always being placed in the most suitable location for their care: known as “outlying” or “boarding”. These patients may be moved around the hospital several times until they reach their “home ward”. Of patients under an Older Person Evaluation Review and Assessment team, who were more likely to be boarding than general medicine patients, those with pre‐existing CI were more likely to be moved 3 or times during their hospital admission (Table 2).^60^ In a further study, boarding patients with dementia and/or delirium had higher mortality within 48 hours of admission.^61^ Although hospital organisational factors result in night‐time bed moves, these were deemed avoidable by 50% of staff surveyed in an audit, and considered detrimental to patient experience.^15^

**Table 2:** Outcomes reflecting differentials in care during hospitalisation^a^

Authors, year	Country	Population	Study design	Main results
“Outlying” and bed moves				
Ranasinghe et al. 2017^60^	Australia	300 patients under older person evaluation review and assessment (OPERA) team, age and sex matched with 300 patients under general physician care	Retrospective matched cohort	Outlying patients and those with 3+ bed moves were more likely to be OPERA patients than general medicine patients, (47.7% vs 31.3%, P < .001 and 22.3% vs 8%, *P* < .001 respectively). Of those with 3+ moves, OPERA patients were more likely to have prior cognitive impairment (OPERA 70.1% vs general medicine 36.4%, *P* = .005). OPERA patients were also more likely to be discharged to residential care or to die than those under general medicine (38.8% vs 9.1%, *P* = .009)
Perimal‐Lewis et al. 2016^61^	Australia	6367 inpatients with dementia and/or delirium	Retrospective descriptive study	“Outlier” patients had higher mortality within 48 hours of admission: OR 1.973 [1.158–3.359], *P* = .012
Royal College of psychiatrists, 2017^15^	UK	Patients with dementia in the acute setting.	National audit	Night‐time bed moves were reported as being avoidable in half of staff surveyed.
Pain and end of life or palliative care				
Sampson et al. 2015^62^	UK	230 patients with an unplanned hospital admission with AMTS <8/10	Prospective cohort	Pain was reported in 38.5% of patients during hospitalisation. Pain at movement and at rest was associated with an increase in the BEHAVE‐AD score (adjusted coefficient 0.20 [0.07–0.32], *P* = .002 and 0.41 [0.14–0.69] *P* = .003 respectively), aggression (adjusted coefficient 0.16 [0.09–0.23], *P* < .001 and 0.16 [0.02–0.30] *P* = .023 respectively) and phobia/anxiety (adjusted coefficient 0.04 [0.01–0.07], *P* = 0.021 and 0.11 [0.04–0.17] *P* = .001 respectively).
Kelley et al. 2008^63^	USA	4 patients aged 70+ with dementia and pain	Prospective case series	Patients with dementia may be unable to describe the characteristics and associated features of their pain, less able to alert staff to the presence of side effects from pain medicines, and unable to discern variations in the level of pain or compare their current pain to their experience of the day or hours before.
Sampson et al. 2006^64^	UK	100 hospital inpatients aged 70+ who died in hospital, 35% with a diagnosis of dementia recorded	Retrospective case‐note review	Patients with dementia had significantly fewer referrals to palliative care (9% vs 25%, *P* = .042) and less frequent prescription of palliative medicines, (28% vs 51%, *P* = .026), than those without. Patients with dementia were more likely to have arterial blood gases checked and to be catheterised, but less likely to have a central line placed. Families were involved in discussing limiting procedures to the same extent (60% vs 53%, *P* = .353).
Afzal et al. 2010^65^	Ireland	75 patients aged 65+ who died in hospital, 24% with dementia	Retrospective case‐note review	Patients with dementia had significantly fewer referrals to palliative care (22.2% vs 62.5%, *P* = .007) less frequent prescription of palliative medicines, (33.3% vs 68.8%, *P* = .017) and carers were less involved in decision making (50.0% vs 87.5%, *P* = .006). There was no difference in the receipt of invasive interventions according to cognitive status.
Formiga et al. 2007^66^	Spain	102 patients aged 65+ who died from dementia (36%) or heart failure in hospital	Case‐note review and carer interviews	No differences between provision of palliative care and withdrawal of drug therapy. In the opinion of the caregiver, adequate symptom control was only present in 46% of patients with dementia, and patients experienced uncontrolled pain and dyspnoea in 13.5% and 51.5% respectively
Formiga et al. 2006^67^	Spain	293 patients aged 65+ who died from dementia (46%), heart failure, or COPD in hospital	Retrospective case‐note review	Rates of drug withdrawal in end‐of‐life patients with dementia in hospital was higher than those with COPD (*P* < .01) or heart failure (*P* < .002)
Aminoff et al. 2005^68^	USA	71 patients with end‐stage dementia, admitted to a geriatric ward in a general hospital	Prospective cohort	The mini suffering state examination scale increased during hospitalisation from 5.62 ± 2.31 to 6.89 ± 1.95 (*P* < .001). 63.4% and 29.6% of patients died with a high and intermediate level of suffering respectively with only 7% dying with a low level of suffering.
Inappropriate catheterization				
Hu et al. 2015^69^	Taiwan	321 patients aged 65+ with a urinary catheter placed during first 24 hours of hospital admission	Prospective cohort with propensity‐matched analysis	Inappropriate catheterisation was defined as NOT meeting 1 of the 6 criteria: Neurogenic bladder dysfunction (where intermittent catheterisation is not possible), urinary retention or bladder outlet obstruction, medication instillation or bladder irrigation, conditions warranting accurate measurement of urinary output, perioperative management, open sacral or perineal wounds with a need for urinary diversion in incontinent patients. Patients with CI (measured by SPMSQ) were more likely to be inappropriately catheterized than those with no CI (65.3% vs 52.6%; *P* = .02), with the rationale of “convenience of care” being reported in almost 50% of cases and leading to a greater decline in ADLs during admission.

a Papers reporting on 1 outcome are repeated as necessary in the other tables of this paper.

#### 3.2.2 Pain and end of life or palliative care

Pain may indicate a new infection, injury, or worsening in condition. The prevalence of pain amongst inpatients with CI is estimated at 39 and is associated with increases in the BEHAVE‐AD score, and increased aggression, phobia, and anxiety.^62^ Dementia reduces a patient's ability to describe pain characteristics and changes, thus delaying diagnosis of infections or overtreating with analgesics like opioids, contributing to complications, eg, delirium, bowel problems, and lengthened stay.^63^ There is no current evidence as to whether patients with CI experience more pain during hospitalisation, probably because of difficulties in assessment.

End‐of‐life patients with dementia have fewer referrals to palliative care and have less prescribed palliative medicines, although no differences were found in 1 study comparing patients with terminal dementia to terminal heart failure.^64^, ^65^, ^66^ Whereas invasive interventions were equally utilised in 1 study, arterial blood gas measurement and catheterisation were more frequent for patients with dementia, and central line placement less used in another study.^64^, ^65^ Drug withdrawal rates in hospitalised end‐of‐life patients with dementia were higher than for patients with COPD or heart failure.^67^ In patients with terminal dementia, only 46% had adequate symptom control, with 13.5% experiencing uncontrolled pain and 51.5% dyspnoeic.^66^ In an evaluation of suffering at end of life in patients with dementia using the Mini Suffering State Examination scale, which includes psychological distress, spiritual concerns, and physical pain, only 7% of patients died with the lowest level of suffering, with the majority experiencing significant suffering, highlighting insufficient assessment and palliative treatment.^68^

#### 3.2.3 Inappropriate catheterisation

Catheterisation could indicate deterioration in a person with CI in hospital, a sign of poor care (if inappropriately performed), or reduction in the ability of staff to provide effective care. The presence of CI was related to inappropriate catheterisation in older patients, with “convenience of care” cited in 50% of cases, and led to a greater decline in ADLs during admission.^69^

### 3.3 Mortality in hospital

Of 11 studies comparing mortality in general inpatients with/without dementia, 8 concluded that patients with dementia have an increased risk of death, with estimates varying from adjusted odds ratio (aOR) 1.09 [1.03‐1.16] to aOR 2.1 [1.0‐4.5] (Table 3).^5^, ^55^, ^70^, ^71^, ^72^, ^73^, ^74^, ^75^, ^76^, ^77^ This difference is greater in people >65 years with dementia as compared to older patients (aOR 1.93 [1.55‐2.41]).^71^ Inpatients with COPD and dementia have a higher mortality risk.^58^ Moderate and severe CI was associated with mortality after ICU admission, even adjusting for acuity scores (Acute Physiology and Chronic Health Evaluation II.^78^ A large cohort demonstrated significant differences in mortality for patients with CI but no diagnosis of dementia as compared to patients with no CI (11.8% vs 9.0%), and a further study showed a difference between “all‐cause” CI and no CI (13.6% vs 9.0%).^10^, ^11^ The presence of CI, regardless of dementia, may independently predict in‐hospital mortality, with the highest risk in patients with severe CI.^5^, ^79^

**Table 3:** Mortality in hospital^a^

Authors, year	Country	Population	Study design	Main results
Barba et al. 2012^70^	Spain	45 757 patients admitted from nursing homes to acute hospitals	Retrospective cohort	17.3% of patients died during hospitalisation, 2442 (30.91%) of them in the first 48 hours. Dementia was an independent predictor of mortality: Adjusted odds ratio (aOR) 1.09 [1.03–1.16]
Marengoni et al. 2011^55^	Italy	1332 patients aged 65 and above admitted to general medicine or geriatric wards	Prospective cohort	9.4% of patients with dementia died in hospital, vs 4.9% of patients without dementia. Dementia was associated with in‐hospital death adjusted odds ratio (aOR) 2.1 [1.0–4.5]. Having dementia and at least 1 adverse clinical event during hospitalisation increased mortality; aOR 20.7 [6.9–61.9].
Draper et al. 2011^71^	Australia	253 000 patients aged 50+ admitted to hospital, including 20 793 with dementia.	Retrospective cohort.	Mortality rates higher for people with dementia across all age groups, with a higher risk in the patients aged 50–64. Estimates range from aOR 50 to 64 years: 1.93 [1.55–2.41] to aOR 85+ years [1.09–1.16]. Overall aOR 1.25 [1.20–1.31].
Hsiao et al. 2015^72^	Taiwan	32 649 elderly patients with dementia and 32 649 controls.	Retrospective propensity score‐matched cohort study	Higher in‐hospital mortality rates for people with dementia at 90 days: aOR 1.97 [1.71–2.27]
Sampson et al. 2009^5^	UK	617 patients aged 70+ with an emergency medical admission	Prospective cohort study	Higher mortality rates for people with DSM IV diagnosis of dementia: aOR 2.09 [1.10–4.00]. Increasing mortality rates with reduction in MMSE (increasing severity of cognitive impairment): MMSE 16–23 aOR 1.34 [0.60–3.15]; MMSE 0–15 aOR 2.62 [1.28–5.39]
Guijarro et al. 2010^73^	Spain	>3 million hospital discharge records of patients aged 65+, including *n* = 40 482 with dementia	Retrospective cohort study	Intrahospital mortality rate was greater for patients with dementia compared to those without dementia (19.3% vs 8.7%). Dementia was an independent predictor of mortality: aOR 1.77 [1.72–1.82]
Oreja‐Guevara et al. 2012^74^	Taiwan	41 672 patients aged 65+, including 3487 with dementia, with a hospital admission	Retrospective cohort study	Dementia was associated with an increased risk of hospital mortality: aOR 1.28 [1.10–1.48]
Farid et al. 2013^75^	France	331 acute patients with cardiovascular disease, age 70+	Prospective cohort	Patients with cognitive impairment had increased mortality HR 2.04 [1.32–3.15]
Zuliani et al. 2011^76^	Italy	51 838 patients aged 60+ admitted to hospital, 4466 with a diagnosis of dementia	Retrospective cohort study	Mortality rate 7.8% in patients with no dementia, vs 10.5% in patients with dementia, *P* = .001
Caspe healthcare knowledge systems (CHKS) 2013^77^	UK	UK‐wide hospital episode statistics of people aged 45+	Retrospective analysis	In 2011, standardised excess mortality rate in patients with dementia estimated at 7.5%.
Liao et al. 2015^58^	Taiwan	COPD inpatients with (*n* = 1406)/without dementia (*n* = 5334)	Retrospective cohort study	Increased risk of mortality for patients with (COPD) with dementia vs no dementia: 4.8% vs 2.3%, aOR 1.69 [1.18–2.43]
Bo et al. 2003^78^	Italy	659 inpatients aged 65+ with an ICU admission during hospitalization	Prospective cohort	Moderate‐to‐severe CI (measured with the SPMSQ) was associated with increased mortality (*P* < .001)
Fogg et al. 2017^10^	UK	19 269 acute hospital admissions of 13 652 patients aged 75+	Retrospective cohort study	Patients with cognitive impairment (no dementia diagnosis) and those with a dementia diagnosis have a higher risk of dying in hospital than patients with no cognitive impairment: 11.8% [10.5–13.3] and 10.8% [9.8–11.9] vs 6.6% [6.2–7.0].
Reynish et al. 2017^11^	UK	10 014 emergency admissions of patients aged 65+, including 38.5% with a cognitive spectrum disorder (CSD)—Delirium, dementia, or AMT <8	Prospective cohort study	Higher mortality in patients with cognitive spectrum disorder (CSD) (delirium, known dementia or abbreviated mental test (AMT) <8/10) than those with no CSD: 13.6% vs 9.0%
Marengoni et al. 2013^79^	Italy	1201 inpatients in internal medicine and geriatric wards	Prospective cohort study	Cognitive impairment (measured by short blessed test) was associated with increased mortality, and this association increased as severity of CI increased: Overall OR 3.1 [1.1–8.6]; moderate impairment: OR 2.7 [1.00–7.96], severe impairment: OR 4.2 [1.29–13.78]
Sa Esteves et al. 2016^28^	Portugal	270 male patients aged 65+ admitted to a medical ward	Prospective cohort study	Mortality rates of patients with/without dementia were similar: 12.1% vs 7.1%; *P* = 0.204
Zekry et al. 2011^80^	Switzerland	444 hospitalised patients aged 75+	Prospective cohort	No association between dementia (HR 0.65 [0.26–1.62]), or cognitive impairment (HR 1.08 [0.29–3.99]) and in‐hospital mortality in univariate analyses
Travers et al. 2014^29^	Australia	493 patients aged 70+, with (n = 102) and without (n = 391) dementia	Prospective cohort study	No difference between mortality rates of people with/without dementia: 5% vs 9%, *P* = .58
Avelino‐Silva et al. 2017^41^	Brazil	1409 patients aged 60+ with acute admission to a geriatric ward	Prospective cohort study	Mortality rates were 8% for patients without delirium or dementia, 12% for patients with dementia alone, 29% for patients with delirium alone, and 32% for patients with DSD (Pearson chi‐square = 112, *P* < .001). DSD and delirium alone were independently associated with in‐hospital mortality: Hazard ratios ratios (HRs) of 2.14 [1.33–3.45], *P* = .002 and 2.72 ([1.77–4.18], *P* < 0.001, but o association between dementia and in‐hospital mortality was found in patients who did not experience delirium during hospitalisation: HR 1.69 [0.72–2.30], *P* = .385
Thomas et al. 2013^81^	Various	Prospective studies consisting of persons aged 65 and older that evaluated the association between at least 1 health‐related participant characteristic and mortality within a year in multivariable analysis.	Systematic review, including 28 studies in hospitals	Cognitive function associated with in‐hospital mortality in 6 of 12 studies (50%)
Zekry et al. 2009^82^	Switzerland	435 hospital patients aged 80+	Prospective cohort	There was no association between presence or severity of dementia or cognitive impairment and mortality in multivariate analysis: Patients with dementia: 3.9% vs 6.3% with MCI and 5.8% with normal cognition, *P* = .641. Clinical dementia rating (CDR) 0.5–1: OR 0.83 [0.07–9.59], CDR 2–3: OR 1.28 [1.12–13.52]
Freedberg et al. 2008^83^	USA	Hospitalised patients aged 85+ and above with/without cognitive impairment (100 in each group)	Matched cohort on age and date of admission.	Cognitive impairment was not associated with increased mortality in multivariate analysis: HR 3.99 [0.42–37.90]
Kimata et al. 2008^84^	Japan	Older patients with (*n* = 62) and without dementia (*n* = 1775) with acute myocardial infarction (AMI)	Prospective cohort	Dementia had no association with increased mortality: 17.7% vs 11.1%, *P* = .101
Tehrani et al. 2013^85^	America	631 734 older patients with (*n* = 15 335)/without dementia with AMI	Retrospective cohort.	Dementia was a significant predictor of in‐hospital mortality for hospitalized individuals with AMI: OR 1.22 [1.15–1.29]. However, there was less likelihood of in‐hospital mortality in participants with dementia who received diagnostic catheterisation (OR 0.36 [0.16–0.78] *P* < .001), percutaneous coronary infusion (PCI) (OR 0.57 [0.47–0.70] *P* < .001), OR CABG (OR 0.22 [0.08–0.56] *P* < .001) than in those not receiving interventions.
Grosmaitre et al. 2013^86^	France	255 patients aged 75+ admitted to emergency departments with ST‐segment elevation MI (STEMI), including 39 patients with dementia	Retrospective cohort	Of 39 patients with dementia, 34 (87.2%) had atypical symptoms at presentation, whilst 5 (4.8%) had chest pain. Atypical symptoms were significantly associated with treatment delays, reduced access to potentially lifesaving treatment, and consequently higher mortality rates at 1 month.
Saposnik et al. 2012^87^	Canada	Patients admitted to hospital with stroke: 877 with dementia and 877 without dementia.	Retrospective propensity score‐matched cohort study	No significant difference in mortality at discharge between patients with/without dementia: Risk ratio (RR) 0.88 [0.74–1.05].
Pisani et al. 2005^88^	USA	395 patients age 65+ with an ICU admission during hospitalisation (*n* = 66 with dementia as per modified blessed dementia rating scale)	Prospective cohort	No association between presence of moderate–severe dementia and mortality (21% for patients with dementia vs 25%, *P* = .53), despite higher acute physiology and chronic health evaluation II scores for patients with dementia on admission to ICU (24.9 vs 22.7, *P* = .02) and higher likelihood of having their code status changed to less aggressive (24% vs 14%, *P* = .04).

a Papers reporting on 1 outcome are repeated as necessary in the other tables of this paper.

Studies which have not shown a difference in mortality between people with/without dementia include a stratified analysis by occurrence of delirium, and 1 study excluding patients with sensorial deficits, communication problems, or severe acute illness, ie, a higher mortality risk.^28^, ^29^, ^41^, ^80^ A systematic review concluded that although cognitive function was a predictor of in‐hospital mortality in 6 of 12 studies assessed, assessments of physical function and nutrition were also important in older patients.^81^ In patients aged ≥80, functional status and comorbidities were predictive of poor outcomes, whereas dementia or other CI was not.^82^, ^83^ Studies exploring the contribution of CI to mortality have been adjusted for a range of covariates, eg, functional/nutritional assessments, comorbidities, and laboratory indicators, which influence estimates of effect.

Contradictory findings regarding the contribution of dementia to mortality in patients presenting to hospital with acute myocardial infarction (AMI) could relate to variation in care provision, as patients with dementia report less chest pain and wait longer for treatment, have fewer transfers to intensive or coronary care units, and less frequent provision of invasive interventions.^84^, ^85^, ^86^ Dementia was not found to be associated with hospital mortality in patients with stroke, or those with an ICU admission.^87^, ^88^

### 3.4 Resource utilisation and discharge destination

#### 3.4.1 Length of hospital stay

In most studies, CI or dementia increased length of hospital stay (LOS).^10^, ^11^, ^71^, ^73^, ^77^, ^89^, ^90^, ^91^, ^92^, ^93^, ^94^, ^95^, ^96^, ^97^ (Table 4) Patients with DSD had longer mean LOS than those with dementia or delirium alone.^11^ Concurrent dementia extends stays in older patients with hip fracture^98^ and haemorrhagic peptic ulcer disease.^99^ However, similar LOS was described in 1 article, and comorbidities found more predictive of longer hospital stays in another study.^76^, ^82^ Discharge after the patient is “medically fit”, because of delays in discharge planning or difficulties in organising residential care, contribute to longer LOS in people with CI,^90^, ^100^ in addition to mental and behavioural manifestations, falls, or hospital‐acquired complications.^45^, ^101^, ^102^ LOS was longer in patients with Parkinsonism‐related dementia or vascular dementia than Alzheimer's, and patients with concurrent diabetes mellitus, pneumonia, and fall‐related hip fracture had more hospital stays of >14 days.^103^

**Table 4:** Resource utilisation and discharge destination^a^

Authors, year	Country	Population	Study design	Main results
Length of stay				
Fogg et al. 2017^10^	UK	19 269 acute hospital admissions of 13 652 patients aged 75+	Retrospective cohort study	Length of stay (LOS) in days (median, IQR): Patients with no CI: 6 (11); CI no diagnosis of dementia: 11 (16); diagnosis of dementia: 9 (17)
Reynish et al. 2017^11^	UK	10 014 emergency admissions of patients aged 65+, including 38.5% with a cognitive spectrum disorder (CSD)—Delirium, dementia, or AMT <8	Prospective cohort study	Mean LOS longer in patients with CSD than those with no CI: 25.0 vs 11.8 days (difference 13.2 [11.2–15.3] *P* < .001). Patients with DSD had significantly longer LOS than those with dementia alone (34.3 vs 20.1 days, *P* < .001) or delirium alone (34.3 23.0 days, *P* < .001)
Power et al. 2017^89^	Ireland	143 patients aged 65+ admitted to hospital, 39 dementia, 30 with mild cognitive impairment (MCI), 74 normal cognition	Prospective cohort study	The mean hospital stay was 32.2 days for patients with dementia, 18.2 days with MCI, and 17.0 days with normal cognition. After adjustment, patients with dementia remained in hospital 15.3 days [1.9–18.8] longer than patients with normal cognition (*P* = .047)
Bo et al. 2016^90^	Italy	1568 patients age 65+ admitted to acute geriatric or medical wards	Prospective cohort study	For patients admitted from home (approx. 90% of the sample), delayed discharge occurred in 392 patients, and was independently associated with cognitive impairment: OR 1.12 [1.05–1.19]. Among patients admitted from intermediate or long‐term facilities, lower cognitive impairment was associated with prolonged stay: OR 0.59 [0.39–0.88].
Tropea et al. 2016^91^	Australia	93 300 hospital admissions of patients aged 50+, including 6459 (6.9%) with CI	Retrospective cohort	Patients with CI had a significantly longer adjusted median length of stay compared with the noncognitively impaired group: 7.4 days (IQR 6.7–10.0) vs 6.6 days (IQR 5.7–8.3), *P* < .001
Guijarro et al. 2010^73^	Spain	>3 million hospital discharge records of patients aged 65+, including n = 40 482 with dementia	Retrospective cohort study	Patients with dementia had a longer average duration of hospital stay than those with no dementia: 13.4 vs 10.7 days
Connolly et al. 2015^92^	Ireland	591 619 adult hospital admissions, with 6702 discharges with a dementia record	Retrospective cohort study	The mean length of stay was higher for patients with dementia than those without across the age groups: 65–74: 24.4 vs 8.7 days; 75–84: 26.8 vs 11.0 days; 85+: 23.7 vs 12.8 days.
Wancata et al. 2003^93^	Austria	372 patients aged 60+ admitted to 4 general hospitals	Prospective cohort study	The mean length of stay of patients with dementia with noncognitive symptoms (eg, depression or delusions) was 30.4 days, vs 23.0 days in patients without such symptoms, vs 16.9 days in patients with no cognitive impairment.
Li et al. 2013^94^	China	34 888 patients aged 60+ admitted to a tertiary hospital, including 918 with dementia	Retrospective case–control study	Patients with dementia had a mean LOS of 13 days (standard deviation (SD) 8–20) vs 15 days (SD 11–23) for those without, *P* < .001.
Annear et al. 2013^95^	Australia	4332 hospital admissions of patients aged 55+	Retrospective cohort	Patients with dementia had a median hospital stay of 5 days in both 2013 and 2014, whereas people without had a stay of 2 days in 2013 and 3 days in 2014.
Draper et al. 2011^71^	Australia	409 000 hospitalisations in 253 000 patients aged 50+	Retrospective cohort	The mean length of stay for admissions for people with dementia was 16.5 vs 8.9 days for those without dementia (*P* < .0001)
Briggs et al. 2016^96^	Ireland	69 718 hospital admissions in patients 65+, including 1433 (2%) admissions with a diagnosis of dementia (929 patients)	Retrospective cohort	The mean LOS was 31 days in patients with dementia, as compared to 14.1 days in patients without a diagnosis.
Lang et al. 2006^97^	France	908 patients aged 75+ with an acute admission to hospital	Propsective cohort	Patients with CI were more likely to stay more than 30 days in hospital: OR 2.2 [1.2–4.0], including after adjustment by French diagnosis related groups: OR 7.1 [2.3–49.9]
Caspe healthcare knowledge systems (CHKS) 2013	UK	UK‐wide hospital episode statistics of people aged 45+	Retrospective analysis	In 2011, standardised excess length of stay in patients with dementia estimated at 22.1%.
Holmes 2000^77^	UK	731 patients aged 65+ with a hip fracture admitted to orthopaedic wards	Prospective cohort	Concurrent dementia or delirium significantly decreased the likelihood of timely discharge as compared to patients with no psychiatric diagnosis: Dementia‐OR 0.47 [0.38–0.58]; delirium‐OR 0.53 [0.41–0.68]
Murata et al. 2015^99^	Japan	14 569 patients aged 80+ treated by endoscopic haemostasis for haemorrhagic peptic ulcer disease, including 695 patients with dementia	Retrospective cohort	Patients with dementia stayed an additional 3.12 [1.58–4.67] days in hospital as compared to those without (*P* < .001).
Zuliani et al. 2011^76^	Italy	51 838 patients aged 60+ admitted to hospital, 4466 with a diagnosis of dementia	Retrospective cohort study	Median length of stay 7 days (IQR 4–12) in patients with no dementia, vs 8 days (IQR 5–12) in patients with dementia, *P* = .12.
Zekry et al. 2009^82^	Switzerland	435 hospital patients aged 80+	Prospective cohort	The median length of stay varied from 41.5 days in patients with dementia: 31 days in patients with MCI, and 29 days in patients with normal cognition, *P* < .001. In multivariate analysis, length of stay was not independently related to cognition: Clinical dementia rating (CDR) 0.5–1: OR 2.12 [0.79–5.69] *P* = .134, CDR 2–3: OR 2.15 [0.75–6.22], *P* = .156
Timmons et al. 2016^100^	Ireland	660 inpatients with a diagnosis of dementia and LOS >5 days	National audit—Retrospective chart review, interviews with senior management and ward managers	72% of people of dementia did not have discharge planning initiated within 24 hours of admission, and less than 40% had a plan for discharge recorded in the notes. The LOS was significantly greater for new discharges to residential care than to usual residence: Median 35 vs 10 days, *P* < .001.
Saravay et al. 2004^101^	USA	93 patients age 65+ admitted to hospital	Prospective cohort	Emergence of mental signs and symptoms in patients with CI, dementia, or delirium prior to behavioural disturbance increase LOS
Chen et al. 2011^45^	Australia	408 patients aged 70+ admitted to hospital	Retrospective case control	Cognitive impairment is related to an increased risk of recurrent falls, and patients with recurrent falls are more likely to have a LOS >5 weeks (50.7% of patients with recurrent falls vs 27.2% with a single fall, and 23.2% with no falls, *P* < .001)
Bail et al. 2015^102^	Australia	426 276 overnight hospital episodes in patients aged 50+, matched 1 patient with dementia: 4 patients without dementia	Retrospective cohort study	People with dementia had increased LOS (10.9 vs 7.1 days).
Chang et al. 2015^103^	Taiwan	203 patients aged 65+ with Alzheimer's, vascular dementia, or parkinsonism‐related dementia admitted to hospital at least once over 4‐year period (472 admissions)	Prospective cohort	Of the dementia subtypes, patients with Alzheimer's had the shortest hospital stays (mean 10.2 days), followed by vascular dementia (16.8 days), and then parkinsonism‐related dementia (17.4 days), *P* = .010. The following were independently associated with prolonged hospital stay (>14 days), specifically: Diabetes mellitus: OR 2.7 [1.17–6.66], *P* = .02; pneumonia: OR 11.21 [3.40–37.01], *P* < .001; fall‐related hip fracture: OR 4.76 [1.18–19.29], *P* = .029.
Costs				
Caspe healthcare knowledge systems (CHKS) 2013	UK	UK‐wide hospital episode statistics of people aged 45+	Retrospective analysis	In 2011, additional costs attributed to excess length of stay in patients with dementia estimated at £83.8 million.
Briggs 2016^77^	Ireland	69 718 hospital admissions in patients 65+, including 1433 (2%) admissions with a diagnosis of dementia (929 patients)	Retrospective cohort	The average cost for a patient with dementia was almost 3 times that of a patient with no dementia: £13 832 vs £5404
Tropea et al. 2016^91^	Australia	93 300 hospital admissions of patients aged 50+, including 6459 (6.9%) with CI	Retrospective cohort	CI (defined as dementia or delirium coded during admission) increased costs of hospitalisation by 51%.
Annear et al. 2016^95^	Australia	4332 hospital admissions of patients aged 55+	Retrospective cohort	Costs of a hospital stay for people with the dementia in the winter months of 2013 and 2014 exceeded the costs of patients without dementia by at least 39%
Connolly et al. 2015^92^	Ireland	591 619 adult hospital admissions, with 6702 discharges with a dementia record	Retrospective cohort study	Estimated that the extra length of stay in patients with dementia results in an additional 246 908 hospital days per annum, at a cost of 199 million euros
Murata et al. 2015^99^	Japan	14 569 patients aged 80+ treated by endoscopic haemostasis for haemorrhagic peptic ulcer disease, including 695 patients with dementia	Retrospective cohort	Average additional costs for patients with dementia were 1171 USD on average (95% CI 533.8–1809.5) *P* < .001.
Bail et al. 2015^102^	Australia	426 276 overnight hospital episodes in patients aged 50+, matched 1 patient with dementia: 4 patients without dementia	Retrospective cohort study	Patients with dementia who had complications during hospitalization accounted for 10.4% of hospital episodes, but comprised 22% of the extra costs.
Lane et al. 1998^104^	USA	3109 patients with Alzheimer's disease at end of life	Retrospective cohort	51% died in hospital, where the costs for end‐of‐life care are estimated to be 6 times higher than hospice or home care.
Araw et al. 2003^105^	USA	60 hospitalised patients with end‐stage dementia	Retrospective cohort	Patients with dementia who had received a palliative care consultation reduced the average daily pharmacy cost from 31.16 USD to 20.83 USD (*P* < .003), even though there was an increase in the prescribing (and therefore costs) of analgesics and antiemetics.
Discharge to a nursing or residential care home				
Fogg et al. 2017^10^	UK	19 269 acute hospital admissions of 13 652 patients aged 75+	Retrospective cohort study	Patients with cognitive impairment (no dementia diagnosis) and those with a dementia diagnosis have higher rates of being discharged to a nursing or residential home than patients with no CI: 11.3% and 16.3% vs 3.5%, *P* < 0.001.
Harrison et al. 2017^106^	Scotland	100 adult patients (18+) with an emergency hospital admission from home and discharged to a care home	Retrospective cohort	75% of new discharges to care homes were in people with cognitive impairment—55% with dementia, and 20% with CI (no dementia diagnosis). Interdisciplinary standards should be set to support assessment and appropriate care for these patients.
Power et al. 2017^89^	Ireland	143 patients aged 65+ admitted to hospital, 39 dementia, 30 with MCI, 74 normal cognition	Prospective cohort study	Patients with dementia were less likely to be discharged home (70.5%), as compared to those with normal cognition (88.8%) or MCI (90%)
Zekry et al. 2009^82^	Switzerland	435 hospital patients aged 80+	Prospective cohort	Dementia is an independent predictor of institutionalisation, ie, a new admission to a nursing home or other long‐term care facility, with patients with severe dementia being 4 times more likely to be institutionalised. Rates of institutionalisation were patients with dementia: 20.1%, patients with MCI: 8.3%, normal cognition: 8.2%, *P* = .001 CDR 0.5–1: OR 1.69 [0.45–6.42] *P* = .438, CDR 2–3: OR 4.17 [1.07–16.26], *P* = .040
Caspe healthcare knowledge systems (CHKS) 2013^10^	UK	UK‐wide hospital episode statistics of people aged 45+	Retrospective analysis	In 2011, deficit in the number of people with dementia with nonelective admissions returning to their usual place of residence estimated at 7.1%.
Draper 2011^71^	Australia	253 000 patients aged 50+ admitted to hospital, including 20 793 with dementia	Retrospective cohort	Patients with dementia were more likely to be discharged to a nursing home across the age groups, increasing from 8.2% in 50–64 years to 22.4% in 85+ years.
Harrison et al. 2017^106^	Various	Observational studies of patients admitted directly to long‐term institutional care following acute hospitalisation, where factors associated with institutionalization were reported. 23 studies (354 985 participants)	Systematic review and meta‐analysis	For the 11 studies included in the quantitative synthesis, patients with dementia had an increased odds of institutionalisation: Pooled OR 2.14 [1.24–3.70].
Kasteridis et al. 2016^108^	England	31 120 patients with a primary diagnosis of dementia admitted to hospital and 139 267 patients with dementia admitted for ambulatory care sensitive conditions	Retrospective cohort study	19% of patients with dementia were discharged to a care home, falling to 14% in patients with an ambulatory care sensitive condition
Saposnik et al. 2012^87^	Canada	Patients admitted to hospital with stroke: 877 with dementia and 877 without dementia.	Retrospective propensity score‐matched cohort study	There was no difference in the proportion of patients going home at discharge: 19.6% with dementia, 19.4% without dementia, RR 1.01 [0.84–1.22]
Leung et al. 2010^109^	UK	N/A	Review	Poor, uncoordinated hospital care may contribute to increased rates of nursing home admissions in people with dementia
Wancata et al. 2003^93^	Austria	372 patients aged 60+ admitted to 4 general hospitals	Prospective cohort study	Both cognitive and noncognitive symptoms of dementia, including depression, agitation, and delusions, were significant independent predictors of nursing home placement. Dementia without noncognitive symptoms: aOR 2.28 [1.37–3.79], *P* = .001; dementia with noncognitive symptoms: aOR 3.61 [1.76–7.38], *P* < .001. In patients with dementia, more severe CI and an increased number of noncognitive symptoms increased likelihood of institutionalisation: aOR 2.82[1.10–7.19], *P* = .030 and aOR 1.38 [1.01–1.88] respectively.
Tochimoto et al. 2015^110^	Japan	391 patients with dementia hospitalised for treatment of BPSD	Prospective cohort study (chart review)	Aggressiveness in BPSD at admission was independently associated with not being discharged home: aOR 0.56 [0.36–0.87], *P* = .010
Brindle et al. 2005^111^	UK	N/A	Discussion paper	Whether the wishes of the individual concerned have been met should be considered in discharge planning, as they may differ markedly from those of health care professionals, carers, or relatives, thus promoting choice and person‐centred care.
Royal College of psychiatrists, 2017^15^	UK	Patients with dementia in the acute setting.	National audit	Over one third of patients did not have their consent to a change in residence after discharge, or evidence that a best interests decision making process had taken place, in the case that they lacked capacity. 54% of carer's comments regarding discharge/care transfer said that discharge was unsafe and poorly planned, which may lead to readmissions to hospital because of lack of readiness of support in the discharge location.

^a^Papers reporting on 1 outcome are repeated as necessary in the other tables of this paper.

#### 3.4.2 Costs

Excess costs relating to increased LOS for patients with dementia exceeded £80 million, and dementia estimated to increase the average cost of an admission 3‐fold (UK figures, 2011).^77^, ^96^ Cognitive impairment (dementia or delirium coded during admission) increased costs of hospital stay by 51% in Australia, and 39% for dementia alone.^91^, ^95^ In Ireland, dementia adds 246 908 hospital days per annum, costing €199 million.^92^ Dementia was associated with increased treatment costs of $1171 for endoscopic haemostasis of hemorrhagic peptic ulcer.^99^ Patients with dementia experiencing complications accounted for 10.4% of hospital episodes, and 22% of extra costs.^102^ Large numbers of patients with dementia die in hospital, where costs for end‐of‐life care can be 6 times higher than hospice/home care,^104^ although appropriate management, eg, palliative care consultations, reduces pharmacy costs through prescribing changes.^105^

#### 3.4.3 Discharge to a nursing or residential care home

Patients with CI are frequently discharged to nursing/residential homes.^10^, ^77^, ^82^, ^89^, ^106^ Dementia predicts of institutionalisation (odds ratio 2.14 [1.24‐3.70]), although less so in ambulatory care sensitive conditions.^71^, ^82^, ^107^, ^108^ However, in stroke patients, no difference in discharge disposition was found between patients with/without dementia.^87^ Contributors to nursing home admissions in people with dementia include poor, uncoordinated hospital care, noncognitive symptoms of dementia (eg, depression, agitation, and delusions), and aggression as part of BPSD.^93^, ^109^, ^110^ Discharge planning should include considering the patient's wishes and using multidisciplinary‐informed standards for discharge from hospital to a care home, although in an audit, consent to a change in residence was not recorded in >30% of patients, nor evidence of “best interests” decision making where patients lacked capacity.^15^, ^106^, ^111^ Fifty‐four per cent of carers' comments regarding discharge/care transfer said that discharge was unsafe and poorly planned, which may lead to readmissions because of lack of available support in the discharge location.

## 4 DISCUSSION

It appears that the presence of cognitive impairment (particularly dementia) in older hospitalised patients influences a variety of clinical and health service outcomes. This is replicated globally, within different health care systems and patient populations. Although most studies focus on patients with diagnosed dementia rather than all‐cause CI, an increased risk of poor outcomes, eg, in‐hospital mortality, delirium, longer LOS, and institutionalisation at discharge was common. Higher mortality rates may partly reflect lack of available suitable care at end of life, lack of end‐of‐life care plans, eg, “do‐not‐hospitalise” advance directives, or unnecessary transfers from nursing homes.^70^, ^112^, ^113^, ^114^, ^115^ Delays in organising appropriate discharge contribute to lengthened hospital stays, highlighting that administrative management and linked services required by these patients may impact on final hospital outcome, as more days in hospital may lead to deconditioning, and policy changes to health and social care infrastructure have unforeseen impacts.^116^

Patients with CI are at increased risk of new infections in hospital, decline in functional and nutritional status, behavioural symptoms, and incontinence. These may be considered “intermediate” outcomes, precipitating patient deterioration, for example, CI was associated with mortality only in patients who had at least 1 adverse event in hospital, and dementia associated with mortality only if delirium had occurred.^41^, ^79^ Such adverse clinical events could indicate a “failure to maintain” patients' basic health needs, leading to further deterioration.^117^ A better understanding of how CI precipitates these events, and what can be done to prevent, detect, and reduce their risk, would enable development of better care models and improved patient outcomes. The multifactorial nature of these events requires a multilevel approach at 7 levels of care—patient, task, staff, team, environment, organisation, and institution—to make improvements, and outcomes for hospital dementia care should reflect changes at each of these levels.^118^ Maintaining clinical and functional status of patients may impact on postdischarge outcomes, eg, mortality, short‐term readmissions, institutionalisation within a year, and continued functional decline.^119^, ^120^, ^121^, ^122^ A focus on fundamentals of care, eg, ensuring nutrition, hydration, skin care and mobilisation of patients, and psychological care, may improve intermediate outcomes and reduce in‐hospital and postdischarge decline.

The variety of covariates used for adjustment in the articles and different approaches used to account for the same underlying characteristics (eg, individual diagnostic groups vs Charlson comorbidity score) may explain variability in study conclusions. For example, functional scores were more significant in predicting mortality than dementia in older patients, but few studies investigating the relationship between CI and mortality adjusted for patient function, suggesting residual confounding. The current trend for including frailty assessments in acute hospital care will provide key information, although it will become difficult to disentangle the relative contributions of frailty and CI, as CI comprises part of commonly used frailty assessments. The majority of studies explored associations between patient characteristics at the beginning of hospital admission with a binary outcome during hospitalisation or outcomes at discharge, not accounting for time‐varying covariates, eg, staffing levels and changes in illness acuity or function. Availability of longitudinal data representing day‐to‐day care, or outcomes reflecting care processes, is essential to understand more about modifiable risk factors contributing to poor outcomes.

Staffing levels, knowledge, and skills are a barrier to provision of best‐practice care for people with CI in hospital.^15^, ^123^ However, studies in this review neither included detailed descriptions of staffing levels and skill mix, staff continuity, training and knowledge, and the general hospital environment, nor took account of these in analyses. Outcomes of value in capturing aspects of care, eg, patient experience, may require specific questionnaires or assessments, and are not commonly available. For example, the person‐centred care of older people with CI in acute care scale (POPAC) measures nursing staff best‐practice care processes to identify CI and employment of nursing interventions to meet associated needs, and could be useful in evaluating routine care and service developments such as training, as well as an outcome in research.^124^

No single study included a wide range of care, clinical, and well‐being outcomes. Given the role of intermediate outcomes in influencing catastrophic events such as mortality, a core outcome set for CI focussed on hospital care is required. This could be used to standardise outcomes for interventional and observational studies, improving comparability of studies, and in routine care to improve care quality and enable evaluation of care innovations. Dementia care audits provide a good starting place to develop outcome sets, as they focus on fundamental care that should be in place to prevent negative outcomes. Examples include delirium screening, mobility assessment, nutritional status, pressure ulcers, pain, continence, and functioning,^15^ plus access to services, eg, liaison psychiatry, speech and language, occupational therapy, social work, and continence services, which indicate holistic care.^100^ Assessments used in long‐term institutions such as the quality of life in late‐stage dementia scale^125^ could be useful, as the hospital environment can negatively influence health outcomes, eg, functional independence and quality of life, through a range of processes.^126^

### 4.1 Limitations

Because of the diffuse questions addressed and limited resources, a single reviewer took decisions on study exclusion and data extraction, involving other reviewers in case of ambiguity. Conclusions would be altered substantively only if a number of large‐scale studies had been accidentally omitted, which seems unlikely. Trial registers were not searched for ongoing studies in this area. Non‐English language articles were not included because of translation restrictions. The majority of findings indicate a relationship between CI and outcomes. Although selective publication of significant results is possible, there would have to be several large unpublished studies to substantially change the overview of findings.

## 5 CONCLUSIONS

Whilst it is important to understand the impact of CI on mortality, length of stay, and institutionalisation, improvement of care for these patients requires insight into the precipitating factors for intermediate outcomes, eg, infections, dehydration, and functional decline, during hospitalisation. Extrinsic factors, eg, staffing and environment, need exploration. Core outcome sets which reflect intermediate outcomes in hospital could be developed and used for clinical trials and quality improvements.

## DISCLAIMER

The research was supported by the National Institute for Health Research (NIHR) Collaboration for Leadership in Applied Health Research and Care (CLAHRC) Wessex at Southampton NHS Hospitals Foundation Trust and Portsmouth Hospitals NHS Trust. The views expressed are those of the authors and not necessarily those of the NHS, the NIHR, or the Department of Health and Social Care.
